# 

**Published:** 1885-05

**Authors:** 


					﻿Whole Number,
CCLCCVI
MAY, 1885.
Number io,
Volume XXIV.
THE
BUFFALO
Medical and Surgical Journal?
EDITORS:
THOS. LOTHROP, M.
A. R. DAVIDSON, M. D.,
Published by the Medical Journal Association.
No. B WEST CHIPPEWA ST.
Entered at the Post-Office at Buffalo, N. Y., as Second-class Mail Matter.
				

